# ROS-Triggered Autophagy Is Involved in PFOS-Induced Apoptosis of Human Embryo Liver L-02 Cells

**DOI:** 10.1155/2021/6625952

**Published:** 2021-04-05

**Authors:** Huai-cai Zeng, Bi-qi Zhu, You-quan Wang, Qing-zhi He

**Affiliations:** ^1^Department of Preventive Medicine, School of Public Health, University of South China, Hengyang 421001, China; ^2^Department of Environmental and Occupational Hygiene, School of Public Health, Guilin Medical University, Guilin 541199, China; ^3^The Second Affiliated Hospital of University of South China, Hengyang, Hunan 421001, China

## Abstract

The liver is the primary target organ for perfluorooctane sulphonate (PFOS), a recently discovered persistent organic pollutant. However, the mechanisms mediating hepatotoxicity remain unclear. Herein, we explored the relationship between reactive oxygen species (ROS) and autophagy and apoptosis induced by PFOS in L-02 cells, which are incubated with different concentrations of PFOS (0, 50, 100, 150, 200, or 250 *μ*mol/L) for 24 or 48 hrs at 37°C. The results indicated that PFOS exposure decreased cell activities, enhanced ROS levels in a concentration-dependent manner, decreased mitochondrial membrane potential (MMP), and induced autophagy and apoptosis. Compared with the control, 200 *μ*mol/L PFOS increased ROS levels; enhanced the expression of Bax, cleaved-caspase-3, and LC3-II; induced autophagy; decreased MMP; and lowered Bcl-2, p62, and Bcl-2/Bax ratio. The antioxidant N-acetyl cysteine (NAC) protected MMP against PFOS-induced changes and diminished apoptosis and autophagy. Compared with 200 *μ*mol/L PFOS treatment, NAC pretreatment reversed the increase in ROS, Bax, and cleaved-caspase-3 protein caused by PFOS, lowered the apoptosis rate increased by PFOS, and increased the levels of MMP and Bcl-2/Bax ratio decreased by PFOS. The autophagy inhibitor 3-methyladenine and chloroquine decreased apoptosis and cleaved-caspase-3 protein level and increased the Bcl-2/Bax ratio. In summary, our results suggest that ROS-triggered autophagy is involved in PFOS-induced apoptosis in L-02 cells.

## 1. Introduction

Perfluorooctane sulphonate (PFOS), a recently discovered persistent organic pollutant, is a toxic and bioaccumulative metabolite of various perfluorinated compounds. PFOS is used for industrial, commercial, and household applications, including textiles, fire-fighting foams, and leather, paper, and food packing materials [1]. It has been detected widely in environmental samples [2, 3]. The reported concentration of PFOS in water from the East China Sea was 703 ng/L [4]. In 2019, Zhou et al. reported that plasma concentrations of PFOS in reproductive age women in Shanghai are 187.2 ng/mL [5]. Results from the Duisburg birth cohort showed that there was a significant association between PFOS concentration quartiles and decreases in body mass index at birth [6]. According to data from the National Health and Nutrition Examination Survey 2003–2008, there was a significantly positive association between total cholesterol and PFOS, as well as for serum albumin with perfluorohexanesulphonic acid (PFHxS) and PFOS [7].

Interestingly, accumulating evidence indicates that the liver is the primary target organ for PFOS [8, 9]. PFOS is associated with total bilirubin (a biomarker of liver function) in the general population in the USA [10]. Using the human liver HepaRG cell line, in 2020, Francoa et al. reported that PFOS interferes with phase I and II biotransformation enzymes, which disrupts biotransformation pathway functions [9]. It has been reported that PFOS induces apoptosis in hepatoma Hep G2 cells [11]. L-02 cells treated with 50 mg/L PFOS can induce apoptosis through p-53 and c-myc signaling pathways [12]. *In vivo* studies showed that PFOS increased liver weight and cell size and disrupted lipid metabolic processes, including mitochondrial *β*-oxidation and lipid metabolism in mice and rats [13–15].

Apoptosis induced by PFOS is reportedly associated with several pathways [16–18]. Reactive oxygen species (ROS) are increasingly blamed for pathogenesis related to PFOS-induced apoptosis. PFOS induces apoptosis of the rat cerebellar granule cell layer of the cerebellar cortex through the ROS-dependent protein kinase C signaling pathway [19]. Additionally, PFOS induces apoptosis of lung adenocarcinoma A549 cells through ROS-mediated mitochondrial dysfunction [17]. PFOS also induces ROS generation, mitochondrial membrane potential (MMP) consumption, and apoptosis of spleen cells and thymocytes in mice [20]. These findings suggest that ROS plays a vital role in PFOS-induced cell apoptosis.

Autophagy plays a pivotal role in energy balance and in quality control of the cytoplasm as a self-regulating catabolic pathway. For example, autophagy regulates hepatocyte functions, and its deregulation has been linked to many liver diseases [21]. The functional relationship between apoptosis and autophagy is complex. Autophagy contributes to stress adaptation which suppresses apoptosis, but it can also be an alternative cell death pathway [22]. In general, autophagy blocks the induction of apoptosis, and conversely, apoptosis-related caspase activation can prevent autophagy. However, in special cases, autophagy or autophagy-related proteins contribute to the induction of apoptosis [23]. Autophagy and apoptosis can also occur in the same cell and autophagy before cell apoptosis [24]. PFOS has been reported to alter autophagy in HepG2 cells [25]. Therefore, in the present work, we used the L-02 hepatocyte line originating from healthy human liver tissues [26] to study the relationship between ROS, autophagy, and apoptosis induced by PFOS and to provide experimental evidence for further studies on the hepatotoxicity of PFOS.

## 2. Materials and Methods

### 2.1. Chemicals

Perfluorooctane sulphonate (PFOS; potassium salt, purity ≥ 98%, Sigma-Aldrich, Shanghai, China) was dissolved in dimethyl sulphoxide (DMSO; Sigma-Aldrich, Shanghai, China) and stored at 20°C. N-acetyl-cysteine (NAC), 3-methyladenine (3-MA), and chloroquine (CQ) were purchased from Sigma-Aldrich (St. Louis, MO, USA; A7250-10G, M9281-100MG, and C6628-25G, respectively). Methyl thiazolyl tetrazolium (MTT) was purchased from Amresco (Solon, OH, USA, 0793-5g). A ROS assay kit was purchased from Beyotime Biotech (Nanjing, China, S0033). An AV-FITC/PI kit was purchased from KeyGen Biotech (Nanjing, China, #KGA108). Monodansyl cadaverine (MDC) and rhodamine 123 (Rh123) were purchased from Beyotime Biotech (P6659-20 *μ*g and C2007, respectively). A BCA kit was purchased from Beyotime (Shanghai, China, P0012S). Anti-p62 was purchased from Proteintech (Shanghai, China, 55274-1-AP). Anti-LC3 was purchased from Sigma-Aldrich (Shanghai, China, L8918-200 *μ*L). Anti-Bcl-2 and anti-Bax were purchased from CST (Shanghai, China, #3498 and #5023, respectively). Anticleaved-caspase-3 and *β*-actin were purchased from Santa Cruz, Paso Robles (CA, USA). Horseradish peroxidase- (HRP-) conjugated secondary antibodies and the SuperSignal West Pico Kit were purchased from Thermo (Waltham, MA, USA).

### 2.2. Cell Lines, Culture, and Pretreatment with NAC, 3-MA, and CQ

The L-02 human embryo liver cell line [26] (abbreviated as L-02 cells, Shanghai Center of Cell Culture of the Chinese Academy of Sciences, GNHu 6) was cultured in six-well plates with RPMI-1640 culture medium (Solarbio, Beijing, China) containing 10% newborn calf serum at 37°C in a 5% CO_2_ atmosphere. To study the protective effect of NAC or 3-MA or CQ on PFOS-treated L-02 cells as described in previous reports [27, 28], 1 mmol/L NAC, 20 *μ*mol/L CQ, and 1 mmol/L 3-MA were used to treat the L-02 cells, which were pretreated with 1 mmol/L NAC for 24 hrs, 20 *μ*mol/L CQ for 24 hrs, or 1 mmol/L 3-MA for 4 hrs before being treated with PFOS.

### 2.3. PFOS Exposure and MTT Reduction Assay

The MTT reduction assay was performed to analyze cell viability. The cells were seeded in 96-well plates in RPMI-1640 medium and cultured overnight at 37°C. When the cell density reached about 70%, cells were treated with 0.1% DMSO or different concentrations of PFOS (50, 100, 150, 200, or 250 *μ*mol/L) for 24 or 48 hrs at 37°C. Subsequently, cells were treated with 200 *μ*mol/L PFOS for different times (0, 6, 12, 24, 36, and 48 hrs) at 37°C. The MTT assay was performed according to the manufacturer's instructions [1].

### 2.4. Measuring ROS Levels in L-02 Cells

ROS levels in L-02 cells were determined using a ROS assay kit following the manufacturer's protocol [29]. Briefly, L-02 cells were pretreated with 1.0 mmol/L NAC and then incubated with different concentrations of PFOS for 12 hrs. Next, half a million cells were collected and treated with 2′,7′-dichlorodihydrofluorescein diacetate (DCFH-DA) at 37°C for 20 mins in the dark, and the excess DCFH-DA was removed using D-Hanks. The cells were collected and transferred to a cuvette, and the fluorescence intensity was detected with a Varioskan Flash 3001 UV-Vis spectrophotometer (Thermo Fisher, Waltham, MA, USA) at an excitation wavelength of 488 nm and an emission wavelength of 535 nm.

### 2.5. Measuring Vacuole Levels in L-02 Cells

Autophagic vacuoles/vesicles were observed as the morphological hallmark of autophagy. DMSO alone served as a control group. L-02 cells were pretreated with NAC for 24 hrs or 3-MA for 4 hrs and then exposed to 200 *μ*mol/L PFOS for 12 hrs, and autophagic vacuoles were labelled with monodansyl cadaverine (MDC) according to Sassi et al.'s method [30]. The cells were incubated with 0.05 mmol/L MDC in PBS at 37°C for 10 mins, then washed three times with PBS. The cells were observed and photographed using a Nikon TE 200 inverted fluorescence microscope (Japan) at an excitation wavelength of 380 nm and emission wavelength of 525 nm.

### 2.6. Measurement of Cell Apoptosis

L-02 cells were pretreated with NAC for 24 hrs or 3-MA for 4 hrs and then exposed to 200 *μ*mol/L PFOS for 12 hrs. DMSO alone served as a control group. The L-02 cells were collected to measure the apoptosis rate using an Annexin V-fluorescein isothiocyanate (FITC) apoptosis measurement reagent kit according to the manufacturer's instructions [31]. Annexin V-FITC (5 *μ*L) was incubated with the collected cells in the dark for 30 mins at room temperature. Propidium iodide (PI, 5 *μ*L) was added, and the cells were monitored immediately using flow cytometry with excitation/emission wavelengths of 488/530 nm. The rate of cell apoptosis was calculated as the proportion of apoptotic cells relative to all cells by flow cytometry.

### 2.7. Measurement of MMP

Rh123, a cationic fluorescent dye which can penetrate the cell membrane, was used to estimate MMP based on the methods reported by Park et al. [32]. L-02 cells were treated with 200 *μ*mol/L PFOS for 6 hrs, 12 hrs, and 24 hrs. Before incubation with 200 *μ*mol/L PFOS for 12 hrs, the cells were pretreated with either 1 mmol/L NAC for 24 hrs or 1 mmo/L 3-MA for 4 hrs, respectively. Half a million cells were collected and incubated with 100 *μ*g/L Rh123 for 30 mins at 37°C in the dark. The MMP value was calculated from the fluorescence intensity of Rh123 aggregates at 488 nm of excitation/525 nm of emission wavelength using an F4500 650-60 fluorescence spectrophotometer.

### 2.8. Western Blotting Analysis

At the end of the indicated treatment, the cells were harvested, washed twice with ice-cold PBS, and completely lysed using a protein extraction kit (KeyGen Biotech, Nanjing, China). The cell lysate was centrifuged at 14,000 × g, 4°C for 15 mins, and the supernatant containing raw protein was collected. The concentration of raw protein was quantified using the BCA method. Proteins were separated by SDS-polyacrylamide gel electrophoresis (SDS-PAGE) and transferred to a nitrocellulose membrane. After being blocked with 10% nonfat milk, the blots were incubated with primary antibodies against either p62 diluted at 1 : 1000, LC3 diluted at 1 : 1000, Bcl-2 and Bax diluted at 1 : 1000, cleaved-caspase-3 diluted at 1 : 1000, or *β*-actin (internal control) diluted at 1 : 1000. The blots were then incubated with the appropriate HRP-conjugated secondary antibodies and detected using a SuperSignal West Pico Kit according to the manufacturer's instructions. The expected protein bands were detected using a Bio-Rad ChemiDoc™ MP imaging system. The relative abundance of target protein (normalized to *β*-actin) was measured with the Gel-Pro Analyzer 4.0 software. All results were representative of three independent experiments. The data were expressed as the means ± S.D.

### 2.9. Data Analyses

All experimental data were analyzed using IBM's SPSS 18.0 software and are presented as expressed by the mean ± S.D. [1, 27]. Statistical significance was identified by ANOVA analysis followed by the least significant difference (LSD) test or the Dunnett T3 multiple comparisons test. A *P* value < 0.05 in a two-sided test was considered statistically significant.

## 3. Results

### 3.1. The Effect of PFOS on Cell Viability

**Figure 1:**
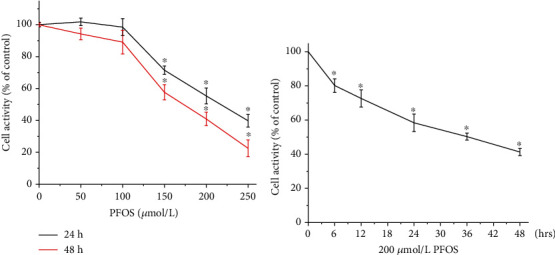
Effects of PFOS on cell viability in L-02 cells. (a) Cells were treated with PFOS for 24 hrs or 48 hrs with different concentrations of PFOS. (b) Cells were treated with 200 *μ*mol/L PFOS for various time periods. Data are presented as the mean ± S.D. (*n* = 3). ^∗^*P* < 0.05 compared with the control group.

L-02 cells were treated with various concentrations of PFOS or DMSO (control) for 24 hrs or 48 hrs, and L-02 cell activities were assessed by the MTT test. PFOS significantly decreased cell viability at higher 150 *μ*mol/L at 24 hrs or 48 hrs (Figure 1(a)). Thus, cells were treated with 200 *μ*mol/L PFOS for different times, and significantly decreased cell viability was observed after 6 hrs following 200 *μ*mol/L PFOS treatment (Figure 1(b)).

### 3.2. The Effect of PFOS on ROS in L-02 Cells

**Figure 2:**
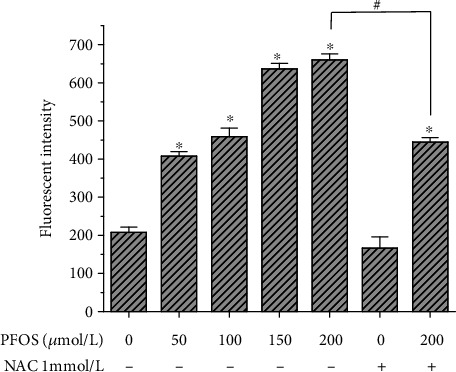
ROS levels in the intracellular microenvironment induced by PFOS. ^∗^*P* < 0.05 compared with the control group, ^#^*P* < 0.05 between the indicated groups.

Fluorescence-based microplate reader analysis was used to quantify PFOS-induced ROS production in L-02 cells. L-02 cells were treated with different concentrations of PFOS (50, 100, 150, and 200 *μ*mol/L) for 12 hrs. Additionally, L-02 cells pretreated with 1 mmol/L NAC were exposed to PFOS at 200 *μ*mol/L for 12 hrs, followed by incubation with DCFH-DA at 37°C for 20 mins in the dark. The results showed that 200 *μ*mol/L PFOS treatment induced a significant increase in ROS generation in L-02 cells (Figure 2). Furthermore, the antioxidant NAC decreased ROS levels induced by PFOS exposure.

### 3.3. Effect of PFOS on MMP

**Figure 3:**
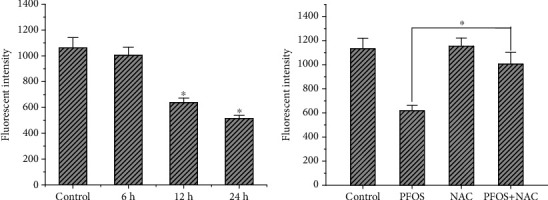
Effect of PFOS on MMP in L-02 cells. MMP was measured using Rh123 staining (*n* = 3). (a) L-02 cells were treated with 200 *μ*mol/L PFOS for 6 hrs, 12 hrs, and 24 hrs. (b) L-02 cells were treated with 200 *μ*mol/L PFOS for 12 hrs, or L-02 cells were pretreated with NAC for 24 hrs and then exposed to 200 *μ*mol/L PFOS for 12 hrs. ^∗^*P* < 0.05 compared with the control group, ^#^*P* < 0.05 between the indicated groups.

In order to explore the effect of PFOS on MMP, L-02 cells were treated with 200 *μ*mol/L PFOS for different times, and Rh123 staining was used to examine MMP. PFOS significantly decreased MMP at the 6, 12, and 24 hr groups compared with the control group (Figure 3(a)). To evaluate the role of oxidative stress in PFOS-induced collapse of MMP, L-02 cells were pretreated with antioxidant NAC before treatment with PFOS. After treatment with 200 *μ*mol/L PFOS for 12 hrs, the MMP was decreased significantly compared with the control (Figure3(b)). Pretreatment of L-02 cells with 1 mM NAC for 24 hrs relieved the PFOS-induced collapse of MMP (Figure 3(b)) which indicated that the PFOS-induced collapse of MMP was related to oxidative stress.

### 3.4. Effect of NAC and 3-MA Pretreatment on Autophagic Vacuoles and Cell Apoptosis Induced by PFOS

**Figure 4:**
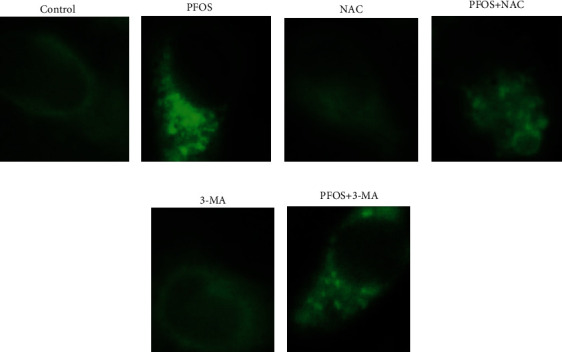
Effects of PFOS, NAC, and 3-MA on monodansyl cadaverine- (MDC-) labelled vesicles: (a) DMSO treatment; (b) 200 *μ*mol/L PFOS treatment; (c) NAC treatment; (d) NAC+200 *μ*mol/L PFOS treatment; (e) 3-MA treatment; (f) 3-MA+PFOS treatment.

It has been reported that MDC is a specific marker for autolysosomes [30]. In L-02 cells being treated with 200 *μ*mol/L PFOS for 12 hrs, compared with DMSO treatment, the number of vacuoles and the fluorescence intensity increased in L-02 cells (Figures 4(a) and 4(b)). Compared with the group treated with 200 *μ*mol/L PFOS alone, the number of vacuoles and the fluorescence intensity decreased slightly in L-02 cells pretreated with NAC for 24 hrs or 3-MA for 4 hrs. (Figures 4(c)–4(f)).

**Figure 5:**
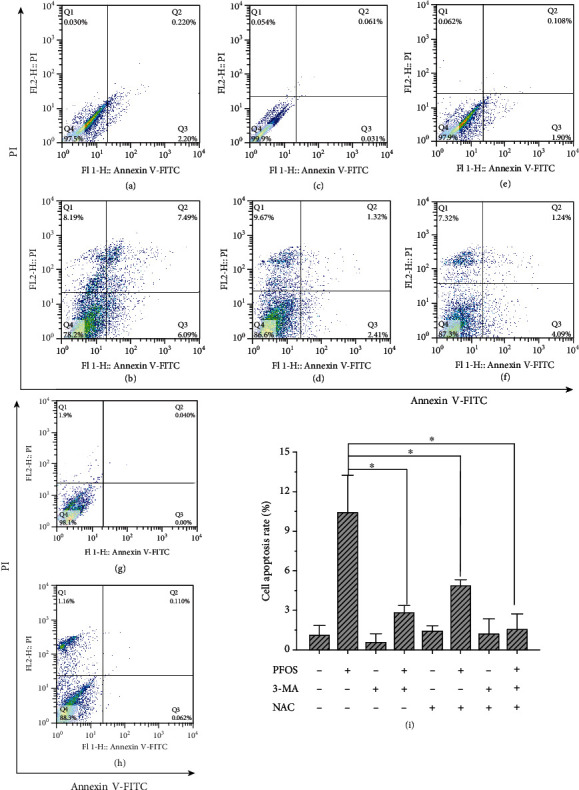
Effects of NAC and 3-MA on PFOS-induced apoptosis in L-02 cells: (a) DMSO treatment; (b) 200 *μ*mol/L PFOS treatment; (c) NAC treatment; (d) NAC+200 *μ*mol/L PFOS treatment; (e) 3-MA treatment; (f) 3-MA+PFOS treatment; (g) 3-MA+NAC treatment; (h) 3-MA+NAC+200 *μ*mol/L treatment; (i) Percentage of apoptotic cells. All data are the mean ± S.D. (*n* = 3). ^∗^*P* < 0.05 between the indicated groups.

Apoptotic cells were determined by flow cytometry with Annexin V-FITC/PI staining. We observed a significant increase in apoptotic cells under exposure to 200 *μ*mol/L PFOS compared with DMSO treatment (Figure 5). Both the antioxidant NAC and the autophagy inhibitor 3-MA decrease the number of apoptotic cells induced by PFOS (Figure 5(i)).

### 3.5. Effects of PFOS on Autophagy- and Apoptosis-Related Protein in L-02 Cells

**Figure 6:**
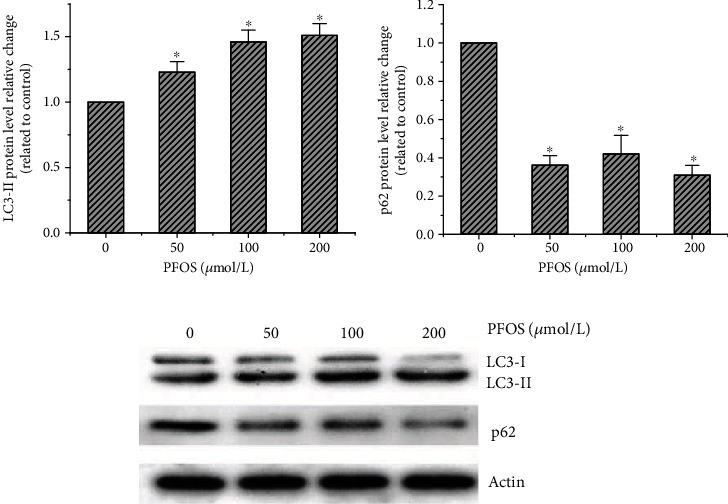
Effect of PFOS on LC3-II/LC3-I and p62 protein levels: (a) LC3-II protein levels; (b) p62 protein levels; (c) representative western blotting lane. Values are represented as the mean ± S.D. (*n* = 3). ^∗^*P* < 0.05 compared with the control group.

**Figure 7:**
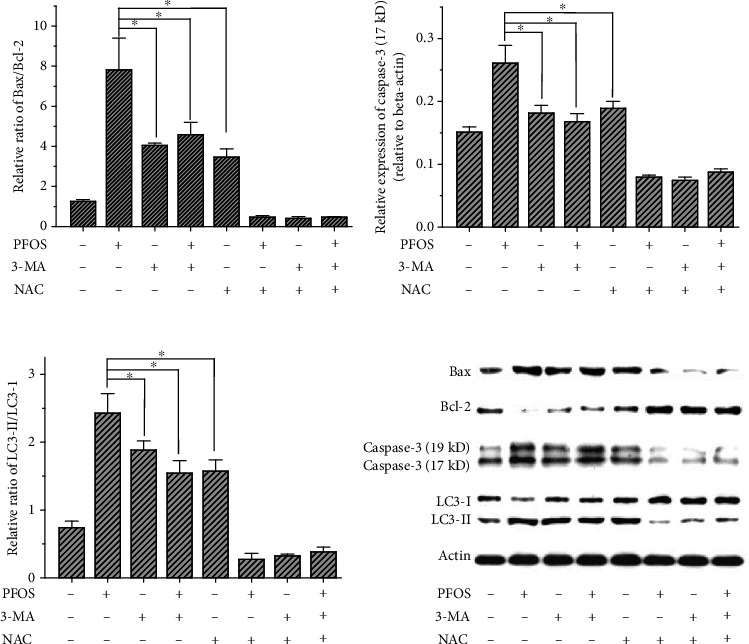
Effect of NAC on the abundance of Bcl-2, Bax, cleaved-caspase-3, and LC3 proteins in L-02 cells following PFOS treatment: (a) relative Bax/Bcl-2 ratio; (b) cleaved-caspase-3 protein (17 kD) level; (c) relative LC3-II/LC3-I ratio; (d) representative western blotting lane. Values were represented as the mean ± S.D. (*n* = 3). ^∗^*P* < 0.05 between the indicated groups.

It has been reported that LC3-II and p62 proteins are markers of autophagy [33], while Bax, Bcl-2, and cleaved-caspase-3 are linked to apoptosis [16]. In order to explore the mechanism of PFOS on L-02 cell apoptosis, the cells were exposed to increasing concentrations of PFOS (50 *μ*mol/L-200 *μ*mol/L) for 12 hrs and autophagy- and apoptosis-related proteins were analyzed by western blot. The results showed that PFOS significantly increased LC3-II levels and decreased p62 levels in a concentration-dependent manner (Figure 6). To further confirm the induction of autophagy, we used chloroquine treatment to assess autophagic flux. CQ pretreatment reduced the LC3-II/LC3-I ratio following PFOS treatment (Figures 7(c) and 7(d)).

In order to investigate the effect of NAC on the abundance of autophagy- and apoptosis-related proteins induced by PFOS, we pretreated cells with NAC and treated the cells with CQ to eliminate the protective role of autophagy via mitochondrial stress. As shown in Figure 7, PFOS significantly increased the abundance of Bax, cleaved-caspase-3, and LC3-II proteins and decreased the level of Bcl-2 protein. NAC reversed the effects to some extent; it reversed the induction of cleaved-caspase-3 and LC3-II by PFOS and decreased the Bax/Bcl-2 ratio.

## 4. Discussion

In the present work, we investigated the effects of PFOS on ROS, MMP, autophagy, and apoptosis with L-02 cells. We found that PFOS exposure decreased cell viability, caused intracellular ROS accumulation, inhibited MMP, and increased autophagy and apoptosis. The antioxidant NAC inhibited ROS generation, recovered the MMP, and decreased autophagy and apoptosis induced by PFOS exposure. The autophagy inhibitors 3-MA and CQ attenuated PFOS-induced apoptosis. These results demonstrated that PFOS induced apoptosis through ROS-triggered autophagy in L-02 cells.

Apoptosis, type *Ι* programmed cell death, is regulated by many genes and signaling pathways such as Bcl-2, Bax, and caspase-3 [16]. Appropriate apoptosis plays a vital role in maintaining normal cell turnover, embryonic development, and immune system function. Conversely, dysregulated apoptosis (either too little or too much) is responsible for many pathogenic conditions including neurodegenerative diseases, ischemic damage, autoimmune disorders, and many types of cancer [34]. Bcl-2 and Bax, two primary regulatory members of the Bcl-2 family, play important roles in apoptosis [34]. Previous studies demonstrated that PFOS upregulates the expression of Bax and downregulates Bcl-2 in the weaned rat heart [16]. The present study confirmed that PFOS induced apoptosis in L-02 cells and apparently increased the Bax/Bcl-2 ratio. Caspase-3 is the key governor of cell apoptosis. In the present study, PFOS also upregulated the expression of cleaved-caspase-3 in L-02 cells and induced apoptosis. Furthermore, PFOS-induced apoptosis was inhibited by the autophagy inhibitor 3-MA and the antioxidant NAC. These results suggest that PFOS-induced caspase-dependent apoptosis was related to oxidative stress and autophagy in L-02 cells.

Autophagy is a complex and highly conserved process, and it is considered a double-edged sword for cell fate. On the one hand, autophagy protects cells against death, but it also mediates cellular demise through type II programmed cell death, depending on specific circumstances [22]. Previous studies suggested that PFOS leads to oxidation-antioxidation imbalance, resulting apoptosis [35] and abnormal autophagy [36], but the relationship remains unclear [20, 37–39]. In the present study, PFOS-induced autophagy in L-02 cells was evident from the appearance of autophagosomes following MDC dying, and this was further confirmed by measuring the turnover of autophagy markers LC3-II and p62 using chloroquine treatment to assess autophagic flux [40]. The results showed that PFOS induced the expression of LC3-II, stimulated autophagic flux, and decreased expression of p62 in a concentration-dependent manner in L-02 cells. The antioxidant NAC reversed the autophagic effect induced by PFOS. This suggests oxidative stress is involved in autophagy induced by PFOS in L-02 cell.

Oxidative stress associated with elevated intracellular ROS levels that cause damage to lipids, proteins, and DNA. Oxidative stress has been linked to many pathologies [41]. Mitochondria are the major source of intracellular ROS [42]. ROS participate in cell signaling and homeostasis at low levels. However, under environmental stimuli, mitochondria are also the target of ROS. Accumulated ROS may inhibit MMP and trigger various downstream effects, including autophagy and apoptosis [43]. Previous studies indicated that PFOS triggered ROS generation in human-hamster hybrid cells [38] and HAPI microglia [44], and ROS played a vital role in PFOS-induced apoptosis [38, 44]. In the present study, we found that PFOS increased intracellular ROS generation in a concentration-dependent manner and caused a significant drop in MMP, which is an important characteristic indicator of aggravated mitochondrial damage. NAC is widely used as an antioxidant to assess the effects of ROS on mitochondrial damage. We found that PFOS-induced ROS generation was inhibited by pretreatment with NAC. In addition, NAC also attenuated PFOS-induced autophagy, apoptosis, and mitochondrial damage and remarkably reversed PFOS-induced cell growth inhibition and attenuated the elevated expression levels of cleaved-caspase-3 and LC3-II. Thus, we speculate that there may be a positive feedback controlling apoptotic signaling. PFOS induced mitochondrial damage and stimulated ROS formation, and ROS accumulation further damaged the mitochondrial membrane, which accelerated autophagy and apoptosis.

## 5. Conclusion

In conclusion, the present study demonstrated that PFOS induced ROS generation, inhibited MMP, and increased apoptosis and autophagy in L-02 cells. Cotreatment with the antioxidant NAC and the autophagy inhibitors 3-MA and CQ decreased ROS generation, protected MMP, and reduced the degree of apoptosis and autophagy induced by PFOS in L-02 cells. Therefore, we speculate that PFOS induces apoptosis of human embryo liver L-02 cells through ROS-triggered autophagy. These findings provide new insight into the molecular basis of PFOS-induced liver toxicity.

## Data Availability

The authors declare that the data supporting the findings of this study are available within the article.
